# Association between chronotype and psychomotor performance of rotating shift workers

**DOI:** 10.1038/s41598-021-86299-8

**Published:** 2021-03-25

**Authors:** Dayane Eusenia Rosa, Luisa Pereira Marot, Marco Túlio de Mello, Elaine Cristina Marqueze, Fernanda Veruska Narciso, Lúcio Borges de Araújo, Cibele Aparecida Crispim

**Affiliations:** 1 Faculty of Medicine, Federal University of Uberlandia, Av. Para, 1720, Bloco 2U, Sala 20, Campus Umuarama, Uberlândia, MG 38405-320 Brazil; 2 Federal University of Minas Gerais, Belo Horizonte, MG Brazil; 3 Catholic University of Santos, Santos, SP Brazil; 4 Faculty of Mathematics, Federal University of Uberlândia, Uberlândia, Brazil

**Keywords:** Health care, Health occupations

## Abstract

It is known that the chronotype potentially mediates the performance and tolerance to work in shifts and that shift rotation is associated with negative effects on psychomotor performance. This study aimed to evaluate the effect of chronotype on psychomotor performance throughout a complete shift rotation schedule. Thirty males working in clockwise rotating shifts from a mining company were evaluated under a real-life condition over the following shift schedule: 2 days of day work, 2 days of evening work and 2 days of night work. The chronotype was determined using the Munich Chronotype Questionnaire adapted for shift workers and the obtained scores were categorized by tertiles (early-type, intermediate-type and late-type). Work performance was evaluated by Psychomotor Vigilance Test (PVT) daily just before shift starts and after shift ends. Sleep duration was evaluated by actigraphy over the whole shift. No isolated effect of the shift or interaction between shift and chronotype was found in the performance variables evaluated. A significant isolated effect of the chronotype showed that the early-type individuals had higher values of pre- and post-work Mean of Reaction Time (MRT) (308.77 ± 10.03 ms and 306.37 ± 8.53 ms, respectively) than the intermediate-type (257.61 ± 6.63 ms and 252.91 ± 5.97 ms, respectively, p < 0.001) and the late-type (273.35 ± 6.96 ms and 262.88 ± 6.05 ms, respectively, p < 0.001). In addition, late individuals presented a greater number of lapses of attention (5.00 ± 0.92; p < 0.05) than early (1.94 ± 0.50, p < 0.05) and intermediate (1.33 ± 0.30, p < 0.001) ones. We concluded that, compared with intermediates, late-type workers had a greater number of lapses of attention on the shift schedule as a whole, while early-type workers showed the highest pre- and post-work MRT. These findings show that the psychomotor performance of rotating shift workers seems to be influenced by the chronotype, but not by the shift rotation.

## Introduction

Social demands around the world require 24-h service production and, consequently, there has been the emergence of shift work^1^. This is defined as any work schedule that does not occur within the usual patterns^2^ and as main characteristic has a continuous 24-h operation^3^. It is estimated that 20% of the workforce worldwide works in shifts^4^. In the United States of America, the percentage has already reached 27%^5^, in Norway 34%^2^ and in Brazil 15%^6^. Shift schedules can be organized in several ways and which could include fixed and rotating shifts^7^. Rotating shifts can differ regarding direction, speed of rotation and timing^8^.

We recently demonstrated that shift rotation is associated with negative effects on psychomotor performance^9^, defined as the ability to maintain and process information for decision making in the workplace^10^. These negative effects include cognitive impairments such as attention drop^11^, memory^12^, decision making^13^, mood alteration^14^, psychological, motor, social and family problems, as well as absenteeism and increased risk of accidents^15,16^. One of the main reasons for such impairments seems to be the deterioration of the sleep pattern^17,18^, which in turn is impacted by the circadian misalignment to which shift workers are exposed^19^. In addition, individual circadian preference, or chronotype, could supposedly be associated with psychomotor performance^20^.

Chronotype governs the preference regarding bedtime and waking hours^21^, and the wake-up time is an important predictor of optimal performance^22^. In this sense, the morning chronotype people find it easier to get up in the morning, perform better in the mornings and fall asleep easily in the early evening. In contrast, evening types report difficulty getting up early, peak activity during late afternoon or evening^23^ and usually go to bed later^24^. Most individuals (60%) are classified as intermediate chronotype, that is, their preferences may vary between morning and evening chronotype^24^.

It is also known that the morning chronotypes seem to be less fatigued in the first part of the day than intermediate and late^22^. A systematic review on this topic evaluated the effect of chronotype on athletic performance and psychophysiological responses to physical activity, through rating of perceived exertion and fatigue scores, of athletes^22^. Authors found that the morning chronotypes had better athletic performances, as measured by race times, in the morning than evening and intermediate chronotypes^22^. On the other hand, intermediate chronotypes seem to be tolerant of the time effect^25^. Vitale et *al*. investigated the sleep quality, daytime tiredness and sleepiness in response to a late-evening high intensity interval training in soccer players classified as intermediate chronotype. The change in the training schedule did not generate significant results in the analyzed variables^25^.

Studies have also showed that the chronotype potentially mediates the performance and tolerance to work in shifts^20,26–29^. In theory, the late chronotype may negatively affect sleep duration^30^ and, possibly, psychomotor performance^20,29^. A study conducted by Van de Vem et al. showed that late chronotypes reported a shorter main sleep duration and more awakenings complaints during morning shift periods^31^. Facer-Childs et al. investigated the impact of chronotype on indices of cognitive and physical performance at different times of day and showed that late chronotypes have a higher daytime sleepiness and perform worse in the morning across all cognitive and physical measures compared to morning chronotypes^27^.

Based on the above information, it would be expected that the interaction between the time of the shift and the chronotype could impact the psychomotor performance of rotation shift workers. However, the association between working hours, chronotype of the individual and psychomotor performance has been little explored in the literature. The few studies that addressed this subject concluded that workers with evening chronotype did not show better psychomotor performance at night^32,33^. In agreement with these findings, Correa et al. (2014) found that morning chronotypes performed equally during the morning and night simulated shifts^34^, but there is still a lack of information on how different chronotypes respond when working hours change rapidly on consecutive days or whether customizing working hours based on the chronotype could facilitate healthy and sustainable employment^31,35^. Further research is necessary on this topic, given that the highly prevalent morbidities in shift workers, such as sleep and mood disorders, seem to be more frequent in late chronotypes^35^.

This study aimed to evaluate the association of chronotype with the psychomotor performance in industrial workers throughout a complete shift rotation schedule. Our secondary objective is to assess the association between chronotype and duration and quality of sleep in the same individuals and condition. We hypothesized that the late chronotypes present a worse psychomotor performance and sleep quality and a shorter sleep duration in the morning shifts than early chronotypes, as well as that early chronotype workers present a better psychomotor performance, sleep quality and a longer sleep duration working during day time compared to night shifts. We also hypothesized that the late chronotype has a worse psychomotor performance in the schedule as a whole, considering that night work occurs in the minority of days.

## Methods

### Participants and ethics

This is an observational and prospective study of industrial workers in a real-life condition. Thirty male shift workers aged between 25 and 52 years agreed to participate and concluded this study. Participants worked at a mining company localized in a city in the Midwest of Brazil, in a rotative shift work schedule. They were included in the study according the following criteria: being a clockwise rotating shift worker (morning-afternoon-night); working in shifts for at least 1 year; working in the operations control panel or leadership position; being able to answer the questionnaires; being able to wear actigraphy monitors and being able to do the Psychomotor Vigilance Test (PVT); had not done trans meridian travel in the 3 months prior to the start of the study; having no diagnosis of sleep-related disorders.

All the shift workers enrolled in the study gave their written informed consent and were informed about the objectives and procedures. The study was approved by the Human Research Ethics Committee of the Federal University of Uberlândia (CAAE: 49689115.0.0000.5152). All methods were carried out in accordance with the ethical guidelines of the Declaration of Helsinki. More about methods and procedure was previously published on Rosa et al. (2019)^9^.

### Procedures

Baseline evaluations took place on the first day of the shift rotation program and involved filling out questionnaires on sociodemographic and health behaviors, as well as the Munich Chronotype Questionnaire (MCTQ). Anthropometric measurements were also collected on this day.

**Figure 1:**
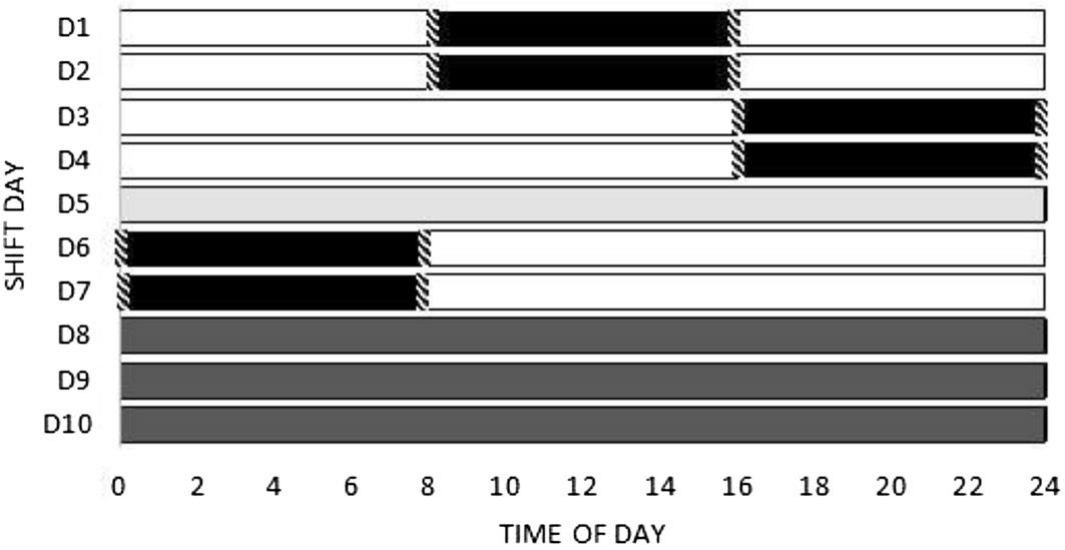
Shift schedule days. Black = work periods. Hatched = PVT tests moments. White = free time on workdays. Light grey = 24 h of rest. Dark grey = free days.

The study protocol started at the first day of the shift schedule adopted by the company. The whole shift schedule comprises: 2 days (D1 and D2) of day shift (08:00–16:00); 2 days (D3 and D4) of evening shift (16:00–00:00); 24 h of rest between evening and night shift (D5); 2 days (D6 and D7) of night shift (00:00–08:00) and three free days (D8, D9 and D10). Evaluations regarding the work performance by PVT were assessed only on work days (D1, D2, D3, D4, D6 and D7). Evaluations regarding sleep duration (actigraphy) and sleep quality (sleep diary) were assessed throughout the whole shift. The PVT was not performed on the 24 h of rest (D5), between the last evening shift and the first one at night, as well as on free days (D8, D9 and D10) (Fig. 1).

### Sociodemographic and health behaviors

A questionnaire was applied aiming to evaluate sociodemographic aspects and health behaviors. The data assessed by the questionnaire were the following: age, marital status, presence of children at home, level of education, years of shift work, frequency of physical activity, alcohol intake, smoking habits, diagnosed diseases and use of medicines.

### Anthropometric variable

A high precision scale of 0.1 kg (Toledo Scale Corp., Toledo, Ohio) and a stadiometer coupled to a scale with an accuracy of 0.1 cm (Toledo Scale Corp., Toledo, Ohio) were used to measure weight and height, respectively. Body mass index (BMI, kg/m^2^) was calculated as the weight (kg) divided by the height squared (m^2^). A BMI < 25 kg/m^2^ was considered eutrophic, ≥ 25 kg/m^2^ and < 30 kg/m^2^ overweight, ≥ 30 kg/m^2^ obesity^34^. These measurements were performed according to standardization methods proposed by Lohman et al. (1988)^36^ and Heyward and Stolarczyk (2000)^37^.

### Munich Chronotype Questionnaire (MCTQ)

The chronotype was determined using Munich Chronotype Questionnaire adapted for shift workers-MCTQ_Shift_^38^, an adapted version from the Munich Chronotype Questionnaire^21^. The Munich Chronotype consists of simple questions about sleep timing (separately on work and free days), allowing the computation of its key parameters: mid-sleep (the midpoint between sleep onset and offset) and sleep duration^21^.

The MCTQ_Shift_ is a validated questionnaire to assess shift workers’ chronotype in rotating schedules, the standard MCTQ items are assessed separately in the morning (M), evening (E), and night (N) shift (in reference to sleep onset before a workday and a free day). Chronotype was determined according the type of rotation shift schedule, with the calculation of "oversleep" for individuals who had a longer sleep duration on free days than on workdays. The MSF_sc_ (Mid-Sleep Time on Free days) is the determination of the chronotype for shift workers considering the following variables: mid-sleep in free days; sleep duration in free days and the "oversleep”^38^.

According to Roenneberg, for statistical analysis, a comparison between the initial, intermediate and final third of the MSFsc distribution should be used, since the MSFsc times is not only specific for age and sex, but also for population^39^. In this sense the data assessed by the MCTQ_Shift_, were ranked by chronotype tertiles and were categorized as “Early-type” (01:30 h–04:41 h), “Intermediate-type” (04:42 h–06:10 h) or “Late-type” (06:11 h–10:21 h).

### Evaluations conducted over shift schedule

#### Actigraphy and sleep diary

The actigraph model used is validated in the Brazilian population^40^. The sleep parameters are calculated by this device through an automatic scoring of wrist activity that provides valuable information about sleep and wakefulness. The software has the optimal algorithms for determining sleep parameters (e.g.: latency, sleep onset/offset, efficiency)^41^. The volunteers were instructed to wear the actigraph on their non-dominant wrist and to fill out a sleep diary with information of their activity during the whole shift schedule^42^. The sleep evaluation on the night shift represents the hours the workers slept at home after the shift ends. Subjective sleep diary data was collected over 24 h throughout the shift (10 days) and have seven questions about sleep timing, sleep latency, number of nighttime awakenings, wakeup method used, daytime napping and an analogic visual scale from 0 to 10 for sleep quality. The data from the analogic visual scale was used to analyze sleep quality, considering 0 the worse rate sleep quality and 10 the best rate. Actigraph and sleep diary data were analyzed simultaneously in order to perform an accurate analysis of the sleep variables, bedtime, wake up time and sleep duration. The researchers doble checked the correct use of the actigraph and sleep diary by cellphone messages and in person during the PVT test times.

#### Psychomotor vigilance performance

PVT is a visual response test involving a luminous stimulus, which displays flashes at intervals from 2 to 10 s on the device screen (irregular intervals) over 5 min^43^. These data make it possible to measure performance in cognitive tasks through variables such as reaction time (RT) and lapses of attention^44^.

In this study, participants were previously "familiarized" with the test/equipment and were instructed that as soon as the visual stimulus appeared they should press the reply button (as fast as possible), located on the right side of the equipment^45^. The test was performed in a quiet illuminated room, individually (only the participant was in the room). The PVT tests were performed at the workplace 5 min just before shift starts and after shift ends. Analyses were performed in the 6 days of work (a complete work schedule). During the 5 min of the test, several stimuli and responses occurred and provide a sampling of reaction time and lapses of attention, which can occur in fatigue conditions (caused by loss of sleep or time in the task)^46^.

The variables analyzed for this study included mean reaction time (MRT) and lapses of attention in pre work and post work. The sum of the number of lapses of attention of pre- and post-work was performed. The lapses are disruptions in performance that typically last between 0.5–15 s^47^. The values obtained were analyzed using the software Microsoft Excel.

### Statistical analysis

ANOVA one way was used to compare the numerical characteristics (age, BMI and sleep duration and quality on free days) between the early-, intermediate- and late-types. Categorical characteristics were compared by the Likelihood ratio test. The paired *t* test was performed in order to compare pre- and post-work values of PVT variables (lapses of attention and MRT) of each shift day. The Generalized linear models (GLM) were used to analyze the effect of chronotype, shift rotation and its interaction on sleep duration, sleep quality and psychomotor performance variables. The analysis was conducted through four statistical models: (1) sleep duration as dependent variable and shift rotation and chronotype as independent variables; (2) MRT-pre work as dependent variable and shift rotation and chronotype as independent variables; (3) MRT-post work as dependent variable and shift rotation and chronotype as independent variables; (4) sum of the number of lapses of attention as dependent variable and shift rotation and chronotype as independent variables. All models were adjusted for age, BMI, years working in shifts and presence of children at home. Normal, Gamma, Inverse Gaussian or Negative binomial (1,5) distribution was chosen considering the smaller Akaike Information Criterion and all pairwise comparisons were performed by Bonferroni test.

Correlations for the potential predictor factors in model were calculated by Generalized Variance-inflation Factor (GVIF < 10). Values lower than 10 were obtained, indicating that there is no multicollinearity among the included variables. Statistical analyses were performed using SPSS version 23.0 (SPSS Inc., Chicago, IL). For statistical significance, α error was set at 5%.

## Results

The one-way ANOVA test and the likelihood ratio showed no significant differences when comparing the characteristics of the groups according to the chronotype. The majority of individuals were married, had children at home, had worked in shifts for 10 years or more, did physical activities at least once a week and were in the overweight range (BMI ≥ 25 to < 30 kg/m^2^) (Table 1).

**Table 1:** Characteristics of the study population according the chronotype (n = 30).

Variables	All	Chronotype			p-value
		Early-type (n = 11)	Intermediate-type (n = 9)	Late-type (n = 10)	
Age (y), mean ± SE	37.2 ± 2.4	38.0 ± 2.5	37.8 ± 2.3	35.8 ± 2.3	0.6478^†^
Children at home, n (%)	19.0 (63.3)	9 (81.8)	5 (55.6)	5 (50.0)	0.2503^‡^
Married or living together n (%)	27.0 (90.0)	11 (100)	8 (88.8)	8 (80.0)	0.1339^‡^
Period of shift work					
> 1 year < 10 years, n (%)	10 (33.3)	2 (18.2)	4 (44.4)	4 (40.0)	
≥ 10 years, n (%)	20 (66.7)	9 (81.8)	5 (55.5)	6 (60.0)	0.3802^‡^
Physical activity—yes, *n* (%)	19.0 (63.3)	6 (60.0)	7 (77.7)	5 (50.0)	0.4408^‡^
BMI (kg/m^2^), mean ± SE	28.43 ± 1.9	28.3 ± 1.9	27.9 ± 1.9	29.03 ± 2.0	0.8194^†^
Overweight, *n* (%)	14.0 (46.7)	5 (45.5)	5 (55.5)	6 (60.0)	0.9039^‡^
Obese, *n* (%)	9.0 (30.0)	4 (36.4)	2 (22.2)	3 (30.0)	
Sleep on free days					
Sleep duration (h), mean ± SE	7.3 ± 0.2	7.6 ± 0.5	7.1 ± 0.3	7.3 ± 0.4	0.7158^†^
Sleep quality, mean ± SE	6.8 ± 0.2	7.2 ± 0.7	6.8 ± 0.3	6.5 ± 0.5	0.7041^†^

Data are expressed as mean ± standard error (SE) or number (percentage). *BMI* body mass index. Overweight BMI ≥ 25 kg/m^2^ and < 30 kg/m^2^; Obesity BMI ≥ 30 kg/m^2^, according to World Health Organization (2000)^29^. ^†^ANOVA One way. ^‡^Likelihood ratio test. Sleep duration data obtained by actigraphy. Sleep quality data obtained by the sleep diary.

Paired *t* test showed that the values of MRT and lapses of attention did not differ statistically pre- and post-work throughout the schedule (p > 0.05).

**Figure 2:**
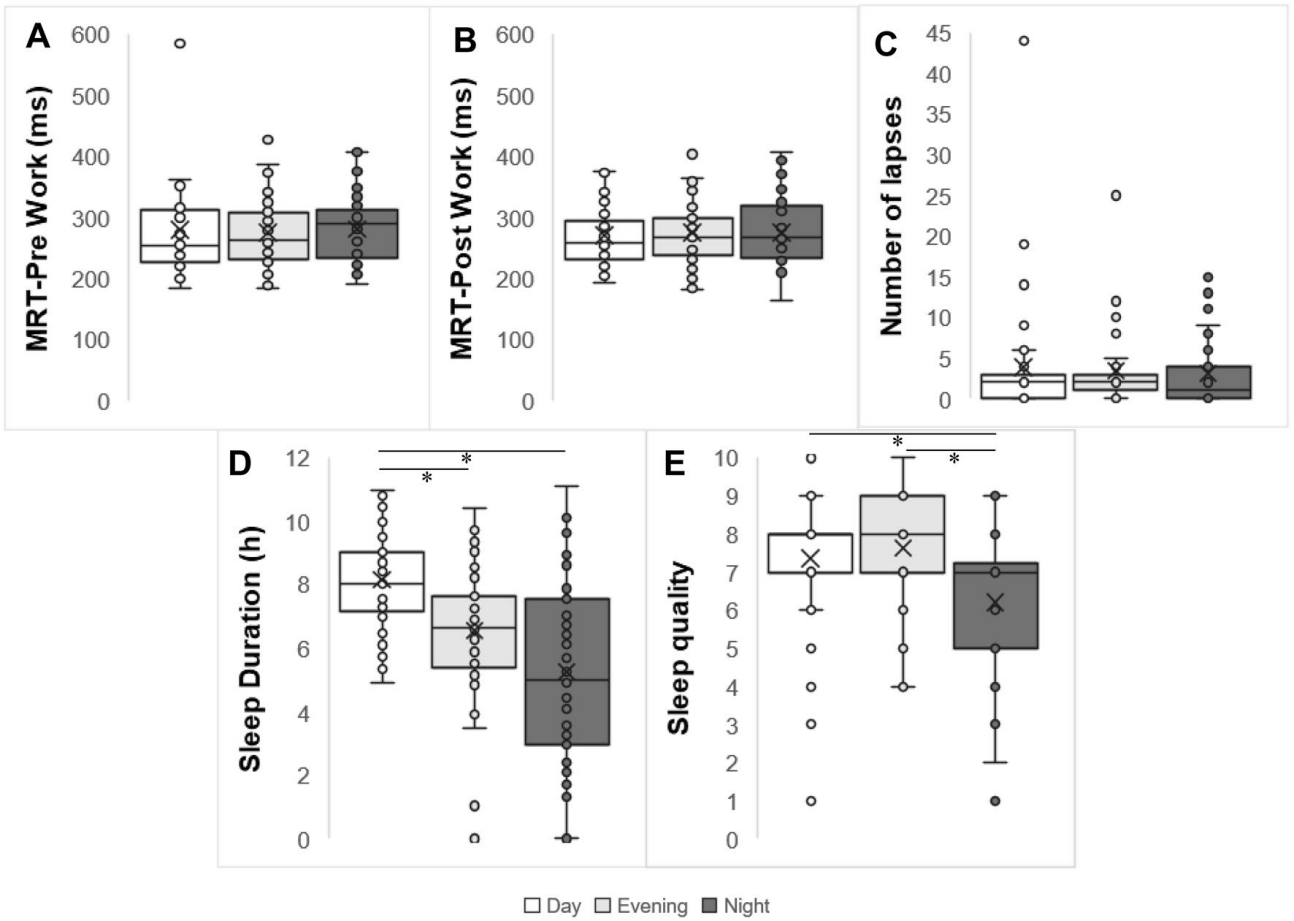
Box plots of the distribution of MRT, number of lapses in attention and sleep duration and quality according to shift. (**A**) MRT pre-work according to shift. (**B**) MRT post-work according to shift. (**C**) Sum of lapses during the shift. (**D**) Sleep duration according to shift. (**E**) Sleep quality according to shift. *Statistical differences, *p* < 0.001. GLM adjusted for age, BMI, years working in shifts and presence of children at home.

The GLM analysis showed a significant difference of sleep duration between shifts. In this sense, the longer sleep duration occurred in day work (8.1 ± 0.3 h) than evening and night shift (6.5 ± 0.3 h and 5.3 ± 0.3 h, respectively; p < 0.01) (Fig. 2D). We did not find significant isolated impact of shift rotation on the PVT variables (MRT-pre work, p = 0.49—Fig. 2A; MRT-post work, p = 0.86—Fig. 2B; lapses of attention, p = 0.88—Fig. 2C). The GLM analysis also showed a significant difference of sleep quality between shifts. The lowest value of sleep quality occurred in night work (6.20) than day work (7.37) and evening work (7.63, p < 0.001) (Fig. 2E).

**Figure 3:**
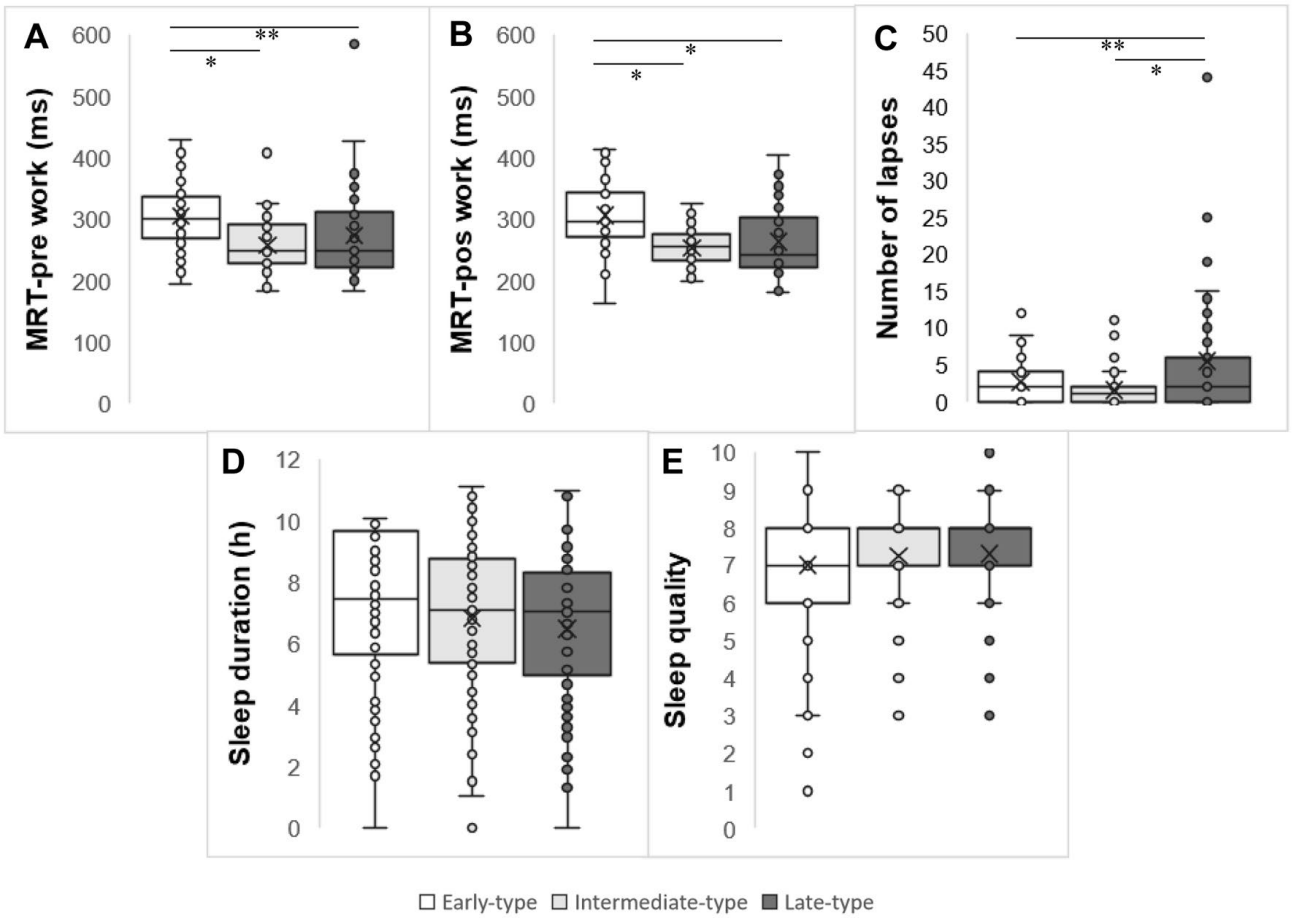
Box plots of the distribution of MRT, number of lapses in attention and sleep duration according chronotypes throughout a complete shift rotation. (**A**) MRT pre work according chronotypes. (**B**) MRT post work according chronotypes. (**C**) Sum of lapses during the shift. (**D**) Sleep duration according chronotypes. (**E**) Sleep quality according chronotypes. Data correspond to mean ± standard error (*n* = 30). *Statistical differences, *p* < 0.001. **Statistical differences, *p* < 0.05. GLM adjusted for age, BMI, years working in shifts and presence of children at home.

Figure 3 presents the differences between chronotypes on mean reaction time, number of lapses of attention, sleep duration, and sleep quality considering all three shifts together. We found the early-type started and ended shift differently than other ones in terms of MRT (Fig. 3A,B), having higher values of MRT-pre work (308.77 ± 10.03) (Fig. 3A) and MRT-post work (306.37 ± 8.53) (Fig. 3B) than the intermediate-type (257.61 ± 6.63 and 252.91 ± 5.97, respectively, p < 0.001) and late-type (273.35 ± 6.96 and 262.88 ± 6.05, respectively, p < 0.001).

The Fig. 3C shows the differences between chronotype on lapses of attention in the schedule as a whole. In general, the late-type group presented higher values of lapses (5.00 ± 0.92) compared to early- and intermediate-types (1.94 ± 0.50 and 1.33 ± 0.30, respectively; p < 0.05). Chronotype groups had no differences regarding sleep duration (p = 0.98) (Fig. 3D) and sleep quality (p = 0.11) (Fig. 3E).

**Figure 4:**
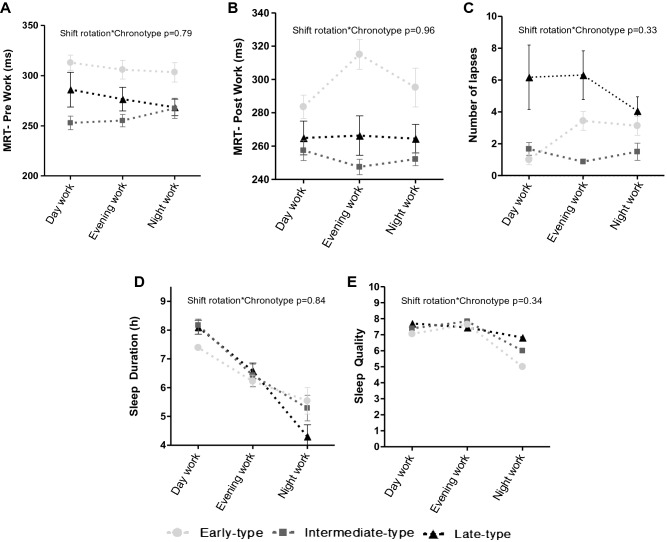
(**A**) Interaction between chronotype and shift rotation on MRT on pre-work. (**B**) Interaction between chronotype and shift rotation on MRT on post-work. (**C**) Interaction between chronotype and shift rotation on sum of lapses during the shift. (**D**) Interaction between chronotype and shift rotation on sleep duration. (**E**) Interaction between chronotype and shift rotation on sleep quality. Data expressed by mean ± standard error of the mean (*n* = 30). GLM adjusted for age, BMI, years working in shifts and presence of children at home.

The GLM test showed that there were no significant interaction between chronotype and work schedules on MRT-pre work (p = 0.79; Fig. 4A), MRT-post work (p = 0.96; Fig. 4B), number of lapses of attention (p = 0.33; Fig. 4C), sleep duration (p = 0.84; Fig. 4D), and sleep quality (p = 0.34; Fig. 4E).

## Discussion

This study showed that the psychomotor performance of rotating shift workers was influenced by the chronotype, but not by the shift rotation per se. We also found that sleep duration and sleep quality were influenced by shift but not by chronotype. These results partially confirm our initial hypothesis. Compared to intermediate workers, late-type workers had a greater number of lapses of attention in the rotating schedule, and the early-type workers showed the highest MRT on pre- and post-work. Taken together, these results show that the MRT is similar between intermediate and late chronotypes, while lapses are not statistically different between early and intermediate types. This means that early and late chronotypes behave differently but are not disadvantaged in the same way compared to intermediates. The evolution of this research area and the confirmation of these findings make it essential for chronotype to be considered when determining the work schedule.

Chronotype has been pointed out as one of the factors that can potentially mediate the tolerance of shift work^20,26–29^. Previous studies on this topic have suggested that evening chronotype workers tend to tolerate night shifts better^20^ and morning individuals present a higher level of circadian misalignment when working the night shift^19^. In this view, we hypothesized that late chronotypes could present a worse psychomotor performance during the morning shifts than early chronotypes, while early chronotype workers could present a better psychomotor performance working during the day than at night. Interestingly, we found that, in terms of mean reaction time, early chronotypes seems to be influenced by the shift rotation as a whole than intermediate types. It is important to point out that early chronotypes from this study may be at a disadvantaged in the timing of test assessments of the psychomotor performance, since they only worked two morning duties (time supposedly preferred by early types) and four supposedly unfavorite duties (two evenings and two nights). In addition to the previous result, the greater number of lapses in attention in late chronotypes than in intermediates ones from the present study seems to be a sign of the flexibility of the intermediate chronotype in a shift rotation schedule.

One of the potential factors impacting the psychomotor performance over the rotational work shifts can be the sleep curtailment^48–52^. The mechanism behind these results is the circadian entrainment caused by fluctuations in bedtime or work schedules time^53^. In addition to the sleep duration, it should be highlighted that the CTS promotes sleep during the dark phase, a period that shift workers usually perform their occupational activities, and promotes wakefulness during the light phase, when shift workers sleep^48,54^. In this sense, the misalignment between circadian rhythms and working hours also negatively impact sleep quality and efficiency^55^. Nevertheless, some individuals may intuitively adapt their behaviors in favor of circadian entrainment caused by rotating shifts, while others, due to chronotype-related characteristics, perform inappropriate or inconsistent sleep hygiene in relation to the light–dark exposure^56^. In the present study, although we found that sleep duration in day work was longer than in evening and night work, we could not identify a difference in sleep duration and quality between chronotype groups in each shift. Vitale et al. demonstrated a greater variation in the sleep pattern over the days in a student population; while the evening types had a reduced sleep quality and quantity compared with the morning and intermediate types during weekdays, the evening types reached the same levels as the other chronotypes during the weekends. Authors concluded that evening types tend accumulate a sleep deficit during weekdays due to social and academic commitments and that they recover from this deficit during free days on the weekend^30^. Interestingly, despite of the same sleep duration in different chronotypes in our study, the intermediate group maintained their levels of psychomotor vigilance better than early and late ones. Other factors such as fatigue^57^, which may also influence psychomotor performance, could justify our results and was not evaluated in the present study.

This study has limitations, such as the limited capacity of extrapolation, considering that we studied a small and entirely male sample. In addition, we only evaluated one shift rotation schedule and only one type of rotation. Thus, we do not know how the psychomotor performance would be affected in other types of shift schedules and rotations. However, we believe that our results are relevant and sufficiently consistent to demonstrate the association between chronotype and psychomotor performance of rotating workers.

## Conclusion

We conclude that the psychomotor performance of rotating shift workers is influenced by the chronotype, but not by the shift rotation. Compared with intermediates, late-type workers had a greater number of lapses of attention on the shift schedule as a whole, while early-type workers showed the highest mean reaction time on pre- and post-work. We also showed effects of the shift, but not of the chronotype or its interaction, on duration and quality of sleep. The longer sleep duration occurred in day work than evening and night shift, while the lowest sleep quality occurred in night work than evening and day. Future studies in this area are important for the chronotype to be considered in determining working hours.
